# The evolution of patient-reported safety concerns during the COVID-19 pandemic within a series of study questionnaires: a multi-method analysis

**DOI:** 10.1093/intqhc/mzaf040

**Published:** 2025-04-29

**Authors:** Isobel Joy McFadzean, Muslim Bilal, Kate Davies, Delyth Price, Thomas Purchase, Anna Torrens-Burton, Denitza Williams, Rhiannon Phillips, Andrew Carson-Stevens, Natalie Joseph-Williams

**Affiliations:** Division of Population Medicine, School of Medicine, Cardiff University, Cardiff CF14 4YS, United Kingdom; Division of Population Medicine, School of Medicine, Cardiff University, Cardiff CF14 4YS, United Kingdom; Division of Population Medicine, School of Medicine, Cardiff University, Cardiff CF14 4YS, United Kingdom; Division of Population Medicine, School of Medicine, Cardiff University, Cardiff CF14 4YS, United Kingdom; PRIME Centre Wales, Division of Population Medicine, School of Medicine, Cardiff University, Cardiff CF14 4YS, United Kingdom; Division of Population Medicine, School of Medicine, Cardiff University, Cardiff CF14 4YS, United Kingdom; Division of Population Medicine, School of Medicine, Cardiff University, Cardiff CF14 4YS, United Kingdom; PRIME Centre Wales, Division of Population Medicine, School of Medicine, Cardiff University, Cardiff CF14 4YS, United Kingdom; Division of Population Medicine, School of Medicine, Cardiff University, Cardiff CF14 4YS, United Kingdom; PRIME Centre Wales, Division of Population Medicine, School of Medicine, Cardiff University, Cardiff CF14 4YS, United Kingdom; Cardiff School of Sport and Health Sciences, Cardiff Metropolitan University, Cardiff CF5 2YB, United Kingdom; Division of Population Medicine, School of Medicine, Cardiff University, Cardiff CF14 4YS, United Kingdom; PRIME Centre Wales, Division of Population Medicine, School of Medicine, Cardiff University, Cardiff CF14 4YS, United Kingdom; Division of Population Medicine, School of Medicine, Cardiff University, Cardiff CF14 4YS, United Kingdom; PRIME Centre Wales, Division of Population Medicine, School of Medicine, Cardiff University, Cardiff CF14 4YS, United Kingdom

**Keywords:** patient safety, patient reported safety concerns, questionnaires, COVID-19

## Abstract

**Background:**

The COVID-19 pandemic had a profound impact on healthcare systems globally, with the potential to aggravate levels of healthcare-associated harm. Due to radical changes within service provision, this period was considered likely to influence patient-reported safety concerns. We aimed to characterise the nature of these safety concerns at different time periods after the first UK lockdown.

**Methods:**

A patient-reported safety concerns module was included within the UK COVID-19 Public Experience (COPE) study surveys at three time points: March/April 2021, September/November 2021, and March/April 2022. Participants were asked whether they had experienced any safety concerns whilst using healthcare services during the previous six months, the nature of the concern(s), and to provide a free-text response to describe it. Free-text data were reviewed to identify reports that met the National Health Service (NHS) definition of a patient safety incident. Descriptive analysis was undertaken to identify incident type, contributory factors, and patient outcomes, followed by thematic analysis of the most frequently reported incidents.

**Results:**

Data from 11,604 completed questionnaires were screened over the three time points, and 1,363 (10.0%) participants reported a safety concern, and 722 (53%) concerns met the definition of a patient safety incident: 262/499 (53%) at 12 months; 215/456 (47.1%) at 18 months; and 245/408 (60.1%) at 24 months. The most frequently reported safety incidents involved access to healthcare professionals (12 months/18 months), and errors managing healthcare appointments (24 months). Prominence of themes fluctuated over time, as the context and policies that influenced the safety reports shifted. For example, geographical limitations on healthcare were evident at 12 months, mitigation from healthcare-associated harm by family members at 18 months, and concerns surrounding healthcare professional and other patient’s behaviour at 24 months.

**Conclusion:**

Healthcare organisations are undoubtedly still undergoing a protracted period of recovery. However, to protect health services from any further threats to functioning, organisations must review patient safety data systems and examine staff perspectives on the issues identified, notably in relation to infection control policies, social distancing, and patient access to health services. Learning from patient-reported experiences and considering how safety incidents are defined would support improvements in patient safety.

## Introduction

Patients face being inadvertently harmed whilst receiving healthcare [1] and it is estimated that 1 in 20 patients are exposed to preventable healthcare-associated harm globally, across primary, secondary, and tertiary care [2]. A reduction in these harms is necessary to lessen the impact on patient morbidity and mortality, with the World Health Organization (WHO), within their global safety action plan, recommending that ‘no-one is harmed in health care…with safe and respectful care, every time, everywhere’ [3].

Healthcare-associated harms can be captured within incident reporting, in which healthcare professionals or allied professionals report events in which patients were, or could have been harmed, whilst receiving healthcare [4, 5]. Similarly, appraisal of medical records, known as case note review, gives insight into care delivery and patient safety concerns [6]. A different, albeit vital source of information into healthcare-associated harm stems from patient-reported safety concerns, as they offer different perspectives to clinician-reported safety incidents and identify events not captured by other methods [7]. Ensuring that their voice is heard, patient-reported safety concerns can be derived from interviews, focus groups, complaints, or questionnaires [8]. The WHO advises that patient-reported experiences and outcomes should guide organisations to prioritise improvements [3], and as patients regularly appraise their care [9], understanding these concerns can enhance patient safety [10].

The COVID-19 pandemic had a profound impact on healthcare systems globally, with potential to aggravate levels of healthcare-associated harm [11]. Whilst there has been significant research into how the pandemic impacted healthcare professionals and patients, with regards to care delivery, within and outside of the UK [12–16], there has been a paucity of research into patient-reported safety concerns within that time, and whether the nature of concerns changed during the pandemic. Due to radical changes within service provision, this period allowed an exploration of the feasibility of collecting patient perspectives to understand their safety concerns, and whether concerns evolved over time.

We aimed to characterise the nature of patient-reported safety concerns at different time periods after the first UK lockdown (March 2020) [17]. Our research question was ‘How did patient-reported safety concerns evolve during the COVID-19 pandemic?’

## Objectives:

- To characterise the nature of patient-reported safety concerns and patient safety incidents during the COVID-19 pandemic.
- To contextualise the concerns in relation to the pandemic to understand the impact of healthcare provision restrictions.
- To make recommendations for future healthcare provision and safer care.

## Methods

### Design

The UK COVID-19 Public Experiences (COPE) study [18] was a prospective longitudinal mixed-method study investigating the psychosocial determinants of health, and wellbeing outcomes during the pandemic [18–20]. This work focuses on its patient-reported safety concern module (Supplementary Appendix 1).

### Setting

Online surveys took place in March/April 2021 (12 months following the first national COVID-19 lockdown), September/November 2021 (18 months post-lockdown), and March/April 2022 (24 months post-lockdown).

Participants were recruited via two methods. Firstly, we used the online platform HealthWise Wales (HWW), which encourages public participation with public health research and helps researchers to find participants for their studies [21]. Members of the public within the HWW registry (∼40 000 people) reside across Wales, with engagement from people from a range of backgrounds, ages etc. to ensure diversity [21]. To support participation from members of the public from across the UK, we also caried out targeted social media campaigns (e.g. Facebook) to encourage recruitment of participants from other areas of the UK in March/April 2020.

Participants were invited to complete optional modules during the 12-/18-/24-month follow-up surveys, sharing some demographical data including shielding status.

### Participants

Inclusion criteria: participants had to be ≥18 years, consented to the study and were able to access the questionnaires online. After being sent initial invitations, non-responders received two reminders.

### Variables

Participant characteristics were collected as part of the main study, and participants within each questionnaire were asked to report safety concerns experienced whilst using healthcare services in the preceding six months, where they happened, and how serious they felt they were, with fixed response items and open free-text responses (Supplementary Appendix 1).

Based on public-partner feedback, we designed a ‘safety concern’ definition (Box 1) in place of the ‘safety incident’ definition predominantly used by healthcare professionals/organisations, as ‘concern’ was more encompassing of the range of outcomes that could be experienced, and denoted near misses and actual incidents.

**Box 1. Definition of safety concern**

Safety concern: “Any event or situation where a patient or other people (including relatives, visitors, NHS staff) might have been harmed whilst accessing National Health Service (NHS) care. This included events or situations where nobody was actually harmed but they could have been, or where someone could be harmed in the future if the concern was not addressed”.

## Data sources/measurement

Utilising the PatIent SAfety (PISA) method [5] which aligns with WHO’s Conceptual Framework for the International Classification of Patient Safety [22], to characterise the nature of the patient responses, we categorised the free-text data. We used a structured framework to apply codes, identifying what happened (incident type), why it happened (contributory factors) and the impact for the patient (outcome) [5], acknowledging that these concerns could involve both physical and psychological harms [23]. Utilising the recursive method of incident analysis allowed us to consider a timeline of events, and create patient vignettes [24]. We then carried out an exploratory descriptive analysis, followed by a thematic analysis to seek patterns and themes within the text.

## Data screening

Free-text responses describing safety concerns were screened by academic General Practitioners trained in patient safety and human factors (J.M. and K.D.), to identify reports that met the patient safety incident definition, ‘any unintended or unexpected incidents which could have, or did lead to harm for one or more patients receiving healthcare’ [25]. To reduce bias, all free-text reports were anonymised and analysed within the same way.

## Study size

All COPE study responses were screened, and confirmed patient safety incident reports were coded by J.M. and K.D. Only information that was explicitly stated within the text was coded, and blank reports were excluded. A random 20% sample of reports were double-reviewed to ensure inter-rater reliability, with a Cohen’s Kappa statistic calculated, aiming for >0.7. Any discrepancies were discussed with the study team, and one senior researcher acted as an arbitrator/arbiter for any disagreements (A.CS.).

## Descriptive/Thematic analysis

Coded data underwent exploratory descriptive statistical analysis to summarise incident frequencies.

To gain a richer understanding of the themes and patterns derived from the text, a thematic analysis focused on a purposive sample at each timeline, using principles from Braun and Clarke [26], and was completed by J.M., with the entire study team iteratively supporting development of themes. Incident reports were read/re-read for familiarisation, and the most frequently reported incidents grouped together, to generate codes and themes to understand salient relationships and patterns.

## Policy context

**Figure 1.**
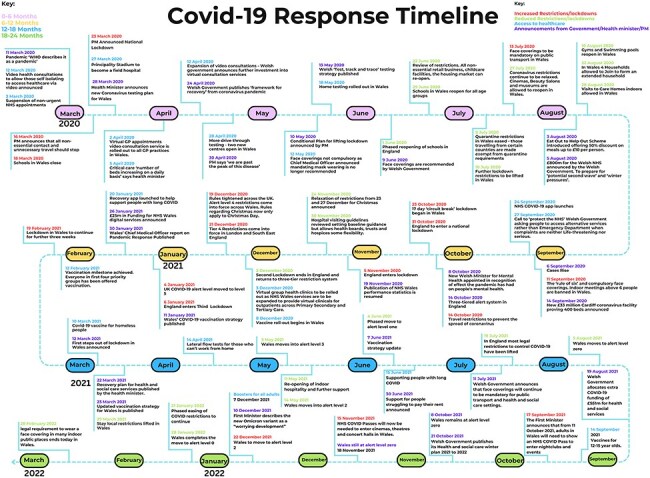
Infographic of rules and regulations throughout the data collection time points [28–30].

Whilst safety concerns are influenced by multiple factors [27], to support the contextualisation of patient responses, COVID-19 related policies within the UK were also summarised and mapped against our findings by academic M.B. (Fig. 1), to support an understanding of contemporaneous safety concerns, and potential responsiveness to the survey.

## Results

**Figure 2.**
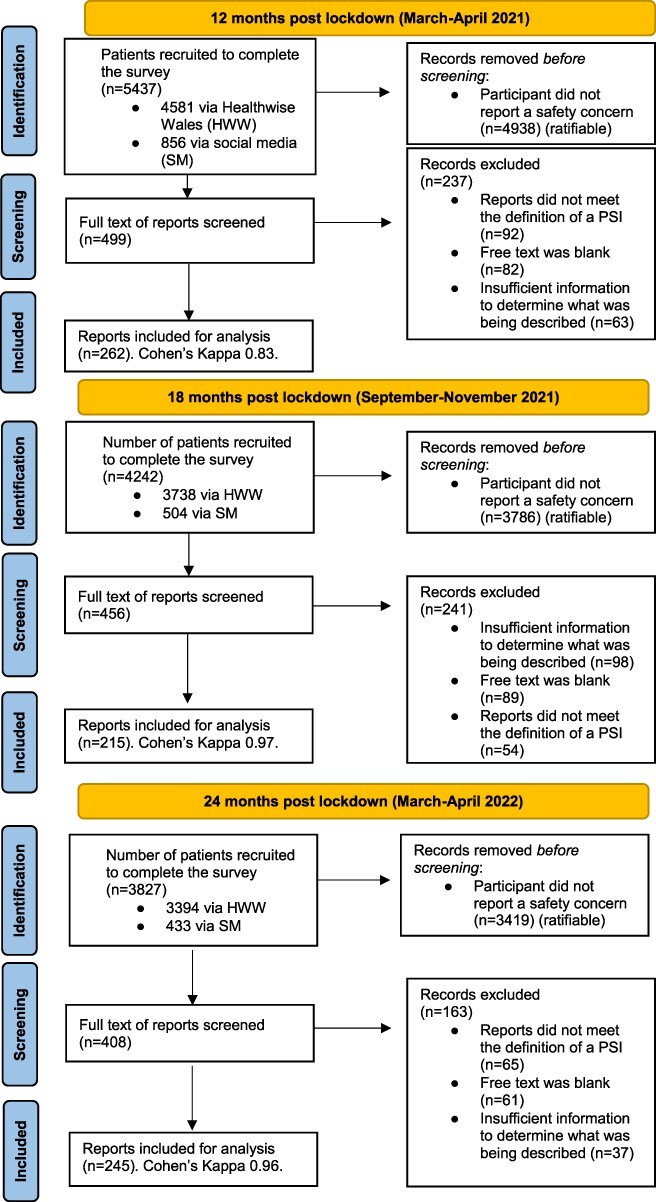
Sample formation at 12, 18, and 24 months.

In total, 11,113 participants consented to participate in the main study [18] and completed the questionnaire 13,604 times in total across all timepoints (see Fig. 2), and 1,848 (16.6%) participants gave consecutive responses to all three surveys.

From these responses, 1,363/13,604 participants (10.0%) reported safety concerns: 499 (36.6%) (12 months), 456 (33.5%) (18 months), and 408 (29.9%) (24 months). The number of responses that met the definition of a patient safety incident were, 262 of 499 (52.5%) (12 months), 215 of 456 (47.1%) (18 months), and 245 of 408 (60.0%) (24 months) with 722 (53.0%) of the concerns in total. Participant demographics for the COPE study and data for the respondents to the safety module can be found in Supplementary Appendices 2 and 3.

### Descriptive analysis

Patients reported difficulties accessing healthcare professionals (HCP) when required, at 12 (70, 27.0%) and 18 months (99, 46%), included accessing secondary care (69 of 169, 40.8%) and General Practitioners (57, 33.7%). They also reported difficulties managing healthcare appointments (63, 29.3%) at 24 months, across primary care (21, 33.3%) and secondary care (7, 11.1%) and in unnamed settings (35, 55.6%). The most frequently reported safety incidents and their contributory factors (CF) are reported in Table 1. All incidents organised by time period can be found in Supplementary Appendices 4–7, with reports containing multiple incidents.

**Table 1.** Most frequently reported patient safety incidents and their contributory factors.

	12 months post-lockdown	18 months post-lockdown	24 months post-lockdown
Top patient safety incident (PSI)	Ability to access healthcare professionals (70)	Ability to access a healthcare professional (93)	Difficulties in managing appointments (63)
Top contributory factors associated with the PSI	Existing chronic conditions (5)	Long wait for service (31)	Long wait for service (9)
	Infection control procedures (4)	Insufficient staff numbers (8)	Family/friend or carer not present (3)
	Child (4)	Comorbidity/housebound patient (7)	-
**Second most frequently reported patient safety incident**	**Errors in managing appointments for healthcare (18)**	**COVID-19 related reports (60)**	**Ability to access a healthcare professional (39)**
Top contributory factors associated with the PSI	Infection control procedures (2)	Infection control policies (48)	Long wait for service (8)
	Equipment (1)	Fear of contracting COVID-19 (29)	Wrong professional carries out task (3)
	Pregnant (1)	Staff behaviour (22)	-
**3rd most frequently reported patient safety incident**	**Communication failures of breakdown in communication (12)**	**Ambulance and A + E waiting times (47)**	**Medications and vaccines (28)**
Top contributory factors associated with the PSI	Infection control procedures (3)	Long wait for service, (18)	Continuity of care (2)
	Interpreter services (1)	Emergency, but no ambulance available (15)	Repeat prescribing (2)
	Protocols inadequate or insufficient (1)	Age/co-morbidity of patient (8)	Patient factors (2)

Most incidents included at least one contributory factor: 66.0% (177) at 12 months, with the most frequent relating to patients detailing infection control policies (101, 58.7%), such as restrictions on where they could attend, and experiencing longer than normal waits for services (18, 10.4%). At 18 months, 73.0% (156), contained at least one CF, with long waits for services (44, 28.2%) and poor continuity of care (40, 25.6%) being the most reported. At 24 months, 64.5% (158) contained at least one CF, with long waits for services (57, 36.0%) and infection control policies (54, 34.2%) featuring prominently again.

Most reports contained insufficient information to determine patient outcomes, with 60.3% (158), being unclear at 12 months, 33% (71) at 18 months and 18.0% (44) at 24 months. Where outcomes were evident, there was predominant deterioration/progression of conditions (32 of 262, 12.2%), or delays in treatment and management at 18 (78 of 215, 36.3%) and 24 months (27 of 245, 11.2%) with the top outcomes reported in Table 2.

**Table 2.** Patient outcomes.

	**12 months**	**18 months**	**24 months**
**First most frequently reported outcome**	General deterioration progression of condition (32)	Delays in treatment and management with an unclear outcome (79)	Delays in treatment and management with unclear outcome (27)
**Second most frequently reported outcome**	Discomfort/pain (18)	Transfer to A + E (8)	Distress (5)
**Third most frequently reported outcom**e	Death (8)	Hospital admission (7)	Additional treatment (2)

There was insufficient data present within the free-text to determine harm severity from the reports.

## Thematic analysis

### Time period 1: 12 months post-lockdown (12 March–13 April 2021)

### Themes:

- Impact of behaviour in seeking healthcare
- Healthcare expectations not being met
- Geographical boundaries exacerbating concerns

Concerns regarding care received were exacerbated by behaviours of others (other people, patients, and healthcare staff) present within healthcare settings, which included failures to adhere to infection control regulations without perceivable repercussions. As per Fig. 1, influences which may have affected public behaviour included ‘Eat out to help out’ schemes in August 2020, a subsequent ‘Circuit breaker’ lockdown in Wales in October 2020 and the launch of the vaccination programme in December 2020. Most concerns related to fears contracting COVID-19, which stopped some participants from seeking medical attention, despite face coverings being mandatory in Wales from July 2020 and social distancing in place:

‘I didn’t go to my next appointment—everyone was too close, and no-one was social distancing. It was really frightening; I didn’t know what to do…’ —Shielding, female participant,

‘…people wandered around not observing social distancing or wearing masks and there was no official to advise those who did not comply. The floors were dirty- my husband and I did not want to stay, but he had chest pain’. —Non-shielding, female participant.

#### Healthcare expectations not being met

During this time, patient expectations of health care, including investigations and appointments, could not be met due to increased demand and physical distancing restrictions in place. Virtual clinics and outpatient appointments were encouraged, but patients experienced significant delays:

‘The tests have still not been conducted and communication is at the point where the NHS do not seem to have any record or resolution for what to do next… the whole end to end care has been poor’. —Non-shielding, male participant

#### Geographical boundaries

Restrictions of travel and geographical boundaries led to safety concerns, especially for participants who lived near geographical borders. Differing restrictions and healthcare availability was seen across different health boards and trusts, and as per Fig. 1, parts of the UK were in lockdown periods at different times:

‘I was worried that a new lump was lymphoma, so was referred to [Welsh hospital] for an USS scan. No appointment came, and when I contacted them, they said it was ‘routine’ and would not be for a long time. I spoke to a specialist, and they brought me to [English] hospital for a scan within a week. England could still take care of me, whereas in Wales, I would still be waiting, and worrying I had terminal cancer’.—Non-shielding, female participant

## Time period 2: 18 months (28 September–3 November 2021)

### Themes:

- Difficulties accessing healthcare
- Poor discharge planning
- Mitigation from harm

### Difficulties accessing healthcare

Participants described how they/loved ones could not access healthcare that they perceived they required, even when acutely unwell. As seen in Fig. 1, virtual health clinics and health services were in place at this time across the UK, but in-person appointments were restricted and long waits for emergency care were widespread.

Whilst some patients preferred online or telephone appointments, others considered this modality to have limited value:

‘My sister came to stay and was very unwell. She was asthmatic and her breathing was really bad, and her oxygen sats were below 90%. Our GP practice said they would not see her as a temporary patient, and she should wait to be seen in out of hours that evening. She couldn’t go to A+E because she had to telephone first, and they said she didn’t need to be seen’. —Shielding, female participant

‘I was only seven weeks from having a heart attack and I got symptoms again. No ambulances were available, and I called 111 and their message said it would be a ∼40 minute wait until someone could answer. When an ambulance finally got to me, they could not get me into [Emergency Department] as there was no space’. —Shielding, female participant

### Poor discharge planning

Some families and patients felt rushed out of hospitals to ‘protect the NHS’, potentially in unsafe conditions. Within Fig. 1, at this time in England, most restrictions had been lifted by July 2021, with vaccinations issued widely, and home testing kits available. Many participants felt that the worst of the pandemic was over, but clinically vulnerable patients still needed support:

‘My stepdad was in the final stages of prostate cancer. He kept being discharged home to my mum who is in her [70s]. This happened nearly five times in a month. He fell in the hospital and broke his hip – and was discharged with this undiagnosed. They also tried to discharge him with Covid to my clinically vulnerable mum, until I stepped in and got social services involved. He died a few weeks later’. —Shielding, female participant

### Mitigation from harm

The participants appeared to become more resilient to restrictions and the impact this had on healthcare provision as the pandemic progressed. They reported mitigating (preventing/lessening) potential harm, via actions taken by themselves or family members, often attempting to treat themselves, or using their own transport when ambulances were not available:

‘Infected tooth – no dentists available. Tried buying a home filling kit to fill the tooth myself’ —Non-shielding, female participant

‘My husband had a stroke, I called 999 as we thought it could have been a heart attack due to pain in his chest. They said there weren’t any ambulances, and they couldn’t send any. I had to drive him to A+E, and he collapsed as we walked in’. –Non-shielding, female participant

## Time period 3: 24 months (22 March–28 April 2022)

### Themes:

- Advocation for healthcare needs
- Concerns regarding staff behaviour
- Impact on quality of life

### Advocation for healthcare needs

As per Fig. 1, here restrictions were lifted, but the impact on routine and non-urgent care was being realized. Patients reported advocating for their healthcare needs:

‘It has become almost impossible to access NHS services and therefore for me to get the diagnosis and treatment I need. You have to be strong to fight your way through, which is often not the case when you are unwell and in need of help… many people are now too traumatised to even have the strength to be able to try again to get help from a doctor’. —Non-shielding, female participant

### Concerns regarding staff behaviour

Patients were less satisfied by the attitudes and behaviour of the healthcare professionals who they felt should have been protecting them, especially with adherence to infection control measures, accompanied with a lack of empathy. Within Wales, facemasks were still required within inside settings, and GP surgeries created their own policies about triage and facemasks:

‘The receptionist in the dentist wasn’t wearing her mask properly—just dangling under her nose…’ —Shielding, female participant and

‘Apparent apathy, and no feeling of guilt keeping us waiting or any sense of urgency by staff…’ —Non-shielding, male participant

### Impact on quality of life:

Many participants reported an overall concern that restrictions and delays receiving healthcare appointments impacted on their wellbeing and overall quality of life. Focus on new conditions such as ‘Long Covid’ and pre-existing conditions was needed.

‘Been waiting for an operation for four years - given a date in March 2020 which was cancelled. His condition has progressed so that his quality of life has deteriorated significantly.. I am concerned about his mental well-being as he is in limbo waiting for surgery and am concerned about how someone with a lower level of health literacy would have coped’. —Non-shielding, female participant

## Discussion

### Principal findings

This study is, as far as we are aware, the first to detail patient-reported safety concerns over 12 months following the first UK COVID-19 lockdown. Up to 10% of participants stated safety concerns, with increased reporting of incidents that met the definition of a patient safety incident at 12 and 24 months.

Participants reported concerns accessing healthcare professionals, and the behaviour of staff/other patients with adherence to infection control measures, consistently across all three time points, indicating ongoing issues.

There was evolution of safety concern reporting over time, with concerns involving the management of healthcare appointments, as they were often cancelled at 12 months and infection control issues at 18 months. There was also an apparent reduction in goodwill as pandemic-related restrictions and protections were removed, with ongoing concerns relating to infection during the later stages of the pandemic at 24 months. Within the reports there was an increase in COVID-19-specific concerns from 18 months onwards, as the public were aware of healthcare-related problems, potentially as they sought care for themselves/loved ones, when services were most severely compromised. This highlights the evolution of safety reporting over time, as participants responded to the changing context of healthcare delivery.

### Interpretation within the context of the wider literature

In the aftermath of the pandemic, healthcare professionals have shared their experiences and perceptions of care globally. Analysis of COVID-19-related incident reports within hospitals in America, have also highlighted delays with investigations, inadequate protocols surrounding the discharge of COVID-19 positive patients, and concerns regarding the behaviour of staff/patients with in relation to infection control measures [11]. In Australia, primary care clinicians, via online surveys, shared similar concerns: about contracting the virus themselves, delays associated with accessing care for acute and long-term conditions, and highlighted the importance of tackling COVID-19 misinformation by ensuring appropriate public health messaging [31].

Healthcare professionals across Australia and England also found administrative issues to be the biggest source of safety concerns, with poor access to healthcare professionals, and problems with diagnoses and assessment [10], whilst Israeli nurses considered the ethical and moral dilemmas associated with the pandemic, agonising whether optimal care can be delivered in such times [32]. These studies emphasise that healthcare professionals recognised the difficulties relating to care delivery during the pandemic, but as it was a period of uncertainty, they often felt unable to overcome the safety issues, which potentially impacted on patient–clinician trust, as well as health outcomes.

There is however an ongoing paucity of information surrounding public-perceived safety concerns related to COVID-19 internationally. Despite this, we expect many patient safety themes will be understood in considerable depth in countries undergoing national-level COVID-19 inquiries. With recognised value placed on the public voice and their diverse opinions and experiences, the COVID-19 inquiry has emulated this in the UK [33]. Whilst official findings of the inquiry are awaited, the campaign ‘Every Story Matters’, encourages members of the public to share their experiences during the pandemic, and aims to support learning and planning for the future [33]. Initial public examples include concerns surrounding shielding, financial implications, and a lack of preparedness by healthcare professionals, leaving many to feel frustrated, fearful, and distrustful of the NHS [33].

Mitigation from harm (reducing the risk of, or preventing harm) by family or friends overall was a novel finding within our study, although this is becoming an important focus in hospitals, such as ‘Martha’s Rule’ and ‘Call 4 Concern’ [34, 35], as well as similar policies internationally that promote patients and families seeking a second opinion. We need to ensure that patients are truly advocated for, and to understand further how healthcare systems use such interventions with carers and families, to overcome problems within the systems themselves.

One of our other themes highlighted implications surrounding geographical boundaries and healthcare delivery. Unsurprisingly, this was identified by other healthcare systems with concerns surrounding rurality. For example, tertiary-centre specialised services within America during the pandemic, identified geographical challenges, with care delivered for patients up to ∼80 miles away [36]. Similarly, in Canada, even pre-pandemic, there were difficulties surrounding the delivery of palliative care across large distances, causing patients to feel isolated when services were not delivered locally [37]. To overcome these issues, Castleden *et al*., recommends the concept of ‘health geography’ which involves healthcare delivery planning, recognition of the distance travelled by patients to receive care services, the appropriateness of locations and consideration of social necessity, i.e. remaining with friends and family [37].

Reporting rates of safety concerns by ∼10% of all participants, is like what has been seen in other research prepandemic. A large longitudinal study, reported that 11% of older patients with long-term conditions in the UK reported safety concerns, and were more likely to experience them if they had multiple long-term conditions and mental health conditions such as depression [38], which was alluded to within our reports. Similarly, studies have shown that complaints data gives similar figures: ∼9% of nearly 2000 reports contained safety reports, usually related to care received and diagnostic errors (irrespective of pandemic and other pressures) [39].

However, universally, there is likely to be an under-reporting of safety concerns, with studies finding ∼5% of participants in England and Australia reported safety incidents [10], with hypotheses about the barriers for patient-reported safety outcomes, including recall bias, which will be explored more thoroughly within other phases of this study, with participant interviews.

Whilst there has been research considering how different sources of safety data overlap, e.g. findings from case note reviews, and related incident reporting [40], we should focus on the type and nature of safety concerns and remember that the patient voice must be at the centre of this work. Incorporating a perspective that is ‘multivoiced’, with focus on all available evidence, e.g. incident reports, medical records, and public-reported concerns would allow for a more complete picture of the pandemic and any related safety concerns.

## Strengths and limitations

These findings were derived from a longitudinal study with a large sample of participants who completed an optional safety module. Whereas most of the participants were from Wales and predominantly female, rates of safety concerns, and safety themes are in keeping with that of other literature. Analysis was carried out rigorously, with high concordance between reviewers across each survey point.

The limitations of surveys are well known [41, 42], including the necessity for participants to have appropriate literacy levels to understand and respond to questions, as well as requiring access to the internet to complete the questions online, which may have limited recruitment. Similarly, the request to describe safety concerns within free-text may have impacted on a willingness to share their experiences. Whilst free-text responses are an invaluable source of information suitable for thematic analysis [43], this may have been reflected within the quality of some of the free-text reports, which subsequently limited analysis. Many participant responses did not contain sufficient information to determine the patient impact, and with an inability to ask further questions or clarify what was meant, this limited the conclusions that could be drawn, especially with contributory factors, which are a key consideration when targeting interventions for improvement.

Further research to explore these patient-reported safety concerns in more depth, using qualitative methods and stakeholder/expert opinion by health boards and policy experts would support an understanding of the nature and determinants of concerns, and may be effective means of gathering further information [44], and identify potential interventions to improve patient safety during pandemics.

### Implications for policy, practice, and research

It is possible that some patient expectations may not have been feasible given the pandemic context, including access to healthcare professionals, availability of healthcare appointments and other healthcare resources during times of emergency provision. This was especially relevant to ambulance, Emergency Department and primary care services, which underwent significant pandemic pressures. Research is ongoing into how to effectively manage patient and public expectations whilst working to address healthcare access during a pandemic [45, 46]. Evidence about effective approaches is still needed around how health services have addressed the backlog of demand. This is important learning that needs to be identified and shared, when available internationally, to help other services resume prepandemic functioning.

In readiness for future planning, a review of discrepancies in care access across geographical boundaries would ensure care equivalence nationally, and that no-one is disadvantaged due to location. Support and guidance from the government and national bodies would assist healthcare organisations to design and implement effective work procedures in times of disasters [47].

Finally, patients require assistance to understand patient safety concepts. Given the high number of reported concerns that did not fit the NHS definition, the NHS would be an appropriate source to help the public to realise that they can/should report these concerns, with attention on preventing any barriers to reporting. Focus on supporting and educating patients to report both negative experiences and patient safety incidents, and to report with as much detail as possible, could support improvements in the reduction of healthcare-associated harm.

## Conclusion

This study offers an important perspective on perceptions of healthcare-associated safety concerns and healthcare-associated harm. NHS organisations need to review their own institutional patient safety incident reporting databases to examine staff perspectives on the issues identified here, notably with infection control policies, social distancing, and patient’s ability to access services.

Focus on the ongoing difficulties surrounding healthcare access is required and patient-reported safety experiences must be further incorporated into safety learning mechanisms, with consideration of how best to support and educate patients to raise safety concerns.

## Supplementary Material

mzaf040_Supp

## Data Availability

Individual-level data from the COPE online survey will not be made publicly available due to data security and ethical considerations. The data provided are of a detailed and sensitive nature. Our public contributors expressed concerns about privacy and security during the development and recruitment stages of this research and did not feel that it was appropriate for individual-level data to be made publicly available. Anonymised data from the COPE study can be made available by the authors on reasonable request, subject to approval from the COPE Study Management Group and Cardiff Metropolitan University Applied Psychology Ethics Panel.
