# Supplementary material for: The evolution of patient-reported safety concerns during the COVID-19 pandemic within a series of study questionnaires: a multi-method analysis
Source: Int J Qual Health Care. 2025 Apr 29;37(2):mzaf040. doi: 10.1093/intqhc/mzaf040 (PMC12510432; doi:10.1093/intqhc/mzaf040)
Supplement: mzaf040_Supp [file mzaf040_supp.zip › suppl_data/Supplementary_files_COPE_18.2.25.docx]

Appendix

**Appendix 1. COPE Survey Safety concern module**

We are interested in any safety concerns that people may have had while accessing healthcare services since [survey specific dates]. This is because health services have been under considerable pressure and are being delivered differently during the pandemic and we would like to understand where there might be areas that could be improved.

Whilst trying to access or receive NHS or private healthcare during the coronavirus pandemic, have you, or someone you care for, experienced something that you thought was a ‘safety concern’?

*Safety concerns can be any event or situation where a patient or other people (e.g. relatives, visitors, NHS staff) might have been harmed while accessing NHS care. This includes events or situations where nobody was actually harmed but they could have been, or where someone could be harmed in the future if the concern is not addressed.*

- Yes – when using NHS services
- Yes -when using private healthcare services
- No
- Don’t know
- Not applicable – I have not used NHS services since [outside of survey dates]
- Rather not say

In which month (or months) did the safety concern happen? Please tick all that apply.

[Survey specific-dates covering the previous six months]

On a scale from 1 (not serious at all) to 10 (extremely serious), how serious do you think your safety concern was?

Not serious at all 1 2 3 4 5 6 7 8 9 10 Extremely serious

In which healthcare setting(s) did the safety concern take place? Please tick all that apply

- COVID-19 testing services
- COVID-19 vaccination services
- GP services (e.g., GP, nurse appointment, health visitor)
- A+E
- Routine outpatient services
- Inpatient services
- Midwifery and Maternity
- District nurse
- Optician
- Pharmacist
- Dentist
- NHS 111
- Other

What did the safety concern(s) relate to? Please tick all that apply.

- Vaccination
- Diagnosis of your problem
- Access to the NHS service you needed
- Tests or procedures that were performed (e.g. blood tests, scans)
- Medication or treatment
- Delay or cancellation of treatment for pre-existing condition
- Communication between you and the healthcare professional(s)
- Communication and co-ordination between different healthcare professionals
- Concerns specific to the coronavirus outbreak (e.g. personal protective equipment)
- Information that was provided to you
- Other

In a few sentences, please tell us a bit more about what happened when you experienced the safety concern, and the impact it has had on you or the person you care for.

Do you think it would have been possible to have stopped this safety concern from happening?

- Definitely yes
- Probably yes
- Probably not
- Definitely not
- Don’t know

**Appendix 2. Demographic data**

| **Demographic and health variables reported at baseline** | | **Baseline (n=11,113)** | | **12 months (n=5437)** | | **18 months (n=4242)** | | **24 months (n=3827)** | |
| --- | --- | --- | --- | --- | --- | --- | --- | --- | --- |
| Gender |  | **n** | **%** | **n** | **%** | **n** | **%** | **n** | **%** |
|  | Male | 3359 | 30.2 | 1786 | 32.8 | 1450 | 34.2 | 1310 | 34.2 |
|  | Female | 7694 | 69.2 | 3629 | 66.7 | 2774 | 65.4 | 2506 | 65.5 |
|  | Missing/rather not say | 60 | 0.6 | 22 | 0.4 | 18 | 0.4 | 11 | 0.4 |
| Age category | 18-30 | 810 | 7.3 | 212 | 3.9 | 128 | 3 | 93 | 2.4 |
|  | 31-40 | 1251 | 11.3 | 437 | 8 | 276 | 6.5 | 235 | 6.1 |
|  | 41-50 | 1459 | 13.1 | 628 | 11.6 | 434 | 10.2 | 393 | 10.3 |
|  | 51-60 | 2352 | 21.2 | 1136 | 20.9 | 874 | 20.6 | 784 | 20.5 |
|  | 61-70 | 3229 | 29.1 | 1865 | 34.3 | 1566 | 36.9 | 1436 | 37.5 |
|  | 71-80 | 1786 | 16.1 | 1037 | 19.1 | 864 | 20.4 | 805 | 21 |
|  | 81+ | 211 | 1.9 | 117 | 2.2 | 97 | 2.3 | 79 | 2.1 |
|  | Missing/rather not say | 15 | 0.2 | 5 | 0.1 | 3 | 0.1 | 2 | 0 |
| Ethnicity | White (Welsh, English, Scottish, Northern Irish, British) | 10,514 | 94.6 | 5174 | 95.2 | 4056 | 95.6 | 3667 | 95.8 |
|  | White other | 316 | 2.8 | 160 | 2.9 | 117 | 2.8 | 103 | 2.7 |
|  | Black/African/Caribbean/Black British | 14 | 0.1 | 4 | 0.1 | 2 | 0 | 1 | 0 |
|  | Asian/Asian British | 58 | 0.5 | 16 | 0.3 | 9 | 0.2 | 10 | 0.3 |
|  | Mixed/multiple ethnic groups | 71 | 0.6 | 30 | 0.6 | 19 | 0.4 | 14 | 0.4 |
|  | Other | 22 | 0.2 | 9 | 0.2 | 8 | 0.2 | 6 | 0.2 |
|  | Rather not say | 118 | 1.1 | 30 | 0.6 | 31 | 0.2 | 26 | 0.6 |
| Marital status | Single | 1638 | 14.7 | 700 | 12.9 | 512 | 12.1 | 460 | 12 |
|  | Married or in civil partnership | 6404 | 57.6 | 3279 | 60.3 | 2576 | 60.7 | 2359 | 61.6 |
|  | Living with partner | 1165 | 10.5 | 492 | 9 | 371 | 8.7 | 314 | 8.2 |
|  | Widowed, divorced or separated | 1722 | 15.5 | 893 | 16.4 | 726 | 17.1 | 643 | 16.8 |
|  | Other | 92 | 0.8 | 36 | 0.7 | 25 | 0.6 | 21 | 0.5 |
|  | Missing/rather not say | 92 | 0.8 | 37 | 0.7 | 32 | 0.7 | 30 | 0.8 |
| Children under 18 years living in household | Yes | 2161 | 19.4 | 810 | 14.9 | 537 | 12.7 | 462 | 12.1 |
|  | No | 8886 | 80 | 4599 | 84.6 | 3684 | 86.8 | 3347 | 87.5 |
|  | Missing | 66 | 0.6 | 28 | 0.5 | 21 | 0.5 | 18 | 0.5 |
| Highest level of education | No college (post 18) education | 3418 | 30.7 | 1540 | 28.3 | 1212 | 28.6 | 1094 | 28.5 |
|  | College (post 18) education | 7458 | 67.1 | 3769 | 69.3 | 2971 | 70 | 2689 | 70.2 |
|  | Missing | 237 | 2.1 | 76 | 1.4 | 59 | 1.4 | 44 | 1.2 |
| Employment status  (NB total > no of participants as some selected more than one category) | Employed full time | 3477 | 31.3 | 1401 | 25.8 | 978 | 23.1 | 886 | 23.2 |
|  | Employed part time | 2124 | 19.1 | 995 | 18.3 | 756 | 17.8 | 641 | 16.7 |
|  | Unemployed | 728 | 6.6 | 330 | 6 | 255 | 6 | 222 | 5.8 |
|  | Retired | 4560 | 41.0 | 2709 | 49.8 | 2272 | 53.6 | 2101 | 54.9 |
|  | In education or training | 399 | 3.6 | 127 | 2.3 | 80 | 2.9 | 61 | 1.6 |
|  | Rather not say | 116 | 1.0 | 33 | 0.6 | 20 | 0.5 | 20 | 0.5 |
| Pre-existing medical condition | Yes | 5607 | 50.5 | 2844 | 52.3 | 2238 | 52.8 | 2022 | 52.8 |
|  | No | 5506 | 49.5 | 2593 | 47.7 | 2004 | 47.2 | 1805 | 47.2 |
| Flu vaccination in last 12 months | Yes | 6089 | 54.8 | 3220 | 59.2 | 2558 | 60.3 | 2339 | 61.1 |
|  | No | 4968 | 44.7 | 2195 | 40.4 | 1672 | 39.4 | 1479 | 38.6 |
|  | Missing | 56 | 0.5 | 22 | 0.4 | 12 | 0.3 | 9 | 0.2 |

**Appendix 3. Demographic data for participants reporting safety concerns**

|  | | **12 months** |  | **18 months** |  | **24 months** |  |
| --- | --- | --- | --- | --- | --- | --- | --- |
|  |  | **N** | **%** | **N** | **%** | **N** | **%** |
| **Gender** | Female | 197 | 75.2 | 172 | 80.0 | 179 | 73.1 |
|  | Male | 62 | 23.7 | 42 | 19.5 | 65 | 26.5 |
|  | Missing | 3 | 1.1 | 1 | 0.5 | 1 | 0.4 |
| **Shielding status** | Don’t know | 34 | 13.0 | 17 | 7.9 | 31 | 12.7 |
|  | No | 201 | 76.7 | 198 | 92.1 | 211 | 86.1 |
|  | Yes | 27 | 10.3 | 21 | 9.8 | 4 | 1.6 |
| **Reporter’s impression of preventability** | Definitely Yes | 138 | 52.7 | 133 | 61.9 | 172 | 70.2 |
|  | Definitely Not | 8 | 3.1 | 7 | 3.3 | 5 | 2.0 |
|  | Don’t Know | 21 | 8.0 | 8 | 3.7 | 12 | 4.9 |
|  | Probably Not | 29 | 11.1 | 9 | 4.2 | 14 | 5.7 |
|  | Probably Yes | 66 | 25.2 | 58 | 27.0 | 46 | 18.8 |
| **Total** | | **262** |  | **215** |  | **245** |  |

**Appendix 4. Nature of patient reported incidents meeting the NHS definition at 12 months**

| **Incident type** | **Example patient narratives** | Frequency |
| --- | --- | --- |
| Ability to access physician/healthcare professional – patient delayed or unable to see/speak to physician or HCP or patient | *He was admitted to hospital with low oxygen and was left in single room.  He didn’t see a nurse for hours.  As it was a weekend they were unable to prescribe him analgesia until a doctor came (it had been hours)…he discharged himself and had to be readmitted the next day.* | 70 |
| Issues managing appointments for healthcare | *Needed to attend A+E for an irregular heartbeat and was then referred by my GP to arrange a 24 hour monitor. Hospital said there were no routine appointments available, which is worrying as I don’t know if this is serious or urgent until I get tested.* | 18 |
| Communication failures or breakdowns of communication | *My mother in law received very poor treatment on several occasions.  There was very poor communication and she was released whilst still very unwell, and discharged to a care home when she was contagious with Covid.* | 12 |
| Insufficient detail | *Visited the hospital for various tests.* | 10 |
| Diagnosis and Assessment | *I had to wait 30 minutes outside of the GP surgery in hot direct sun, whilst in severe pain. I underwent tests but I have heard nothing since, and the pain continues.* | 9 |
| Timeliness of treatment other than medication – not administered in a timely fashion | *My father’s treatment was delayed such that he was so ill he went into hospital. When there, his treatment was further delayed beyond the guidelines, and he died.* | 9 |
| Discharge planning – includes premature discharge and poor discharge planning e.g. patient discharged home when not appropriate or safe to do so | *Father was taken to A+E in an ambulance after collapsing.  He was then discharged from the hospital due to a shortage of beds, which resulted in him having a stroke when he returned home.* | 8 |
| Insufficient treatment / care / monitoring given | *Mum was end of life and the plan was to monitor her oxygen saturations and tweak the oxygen to maintain correct levels. She did not have covid but was moved to a covid ward and they stopped monitoring her oxygen saturations, which I think hastened her end of life.* | 5 |
| Appropriate follow up – incorrect or inefficient transfer of patient for follow-up  e.g. requirements for follow up screening or re-review | *I had an operation which went well, but the aftercare was non-existent. I was not shown how to do the blood-thinning injections, not given a sharps box and was not given the ward phone number in case of problems.* | 4 |
| Delayed diagnosis | *My boyfriend has had emergency eye surgery and this was diagnosed late with limited access to specialists until his retinal tear became a fully detached retina.* | 4 |
| Insufficient assessment - issue during the process of assessing a patient | *My daughter had two large post-partum haemorrhages [PPH] whilst she was at home and I was looking after her and her baby. She was sent home after the first PPH – they told her they were very busy and she was making a fuss about nothing.* | 4 |
| Prescribing medication – wrong medication or wrong dose of medication prescribed or medication not prescribed when appropriate | *GP did not prescribe suitable medication, this was spotted by the pharmacist and confirmed by the out of hours GP.* | 4 |
| Dispensing medication orders – problem within the process of delivering a medication order or inappropriate medication order | *Was given the completely wrong medication by pharmacy. It was prescribed correctly, labelled correctly but the wrong item was dispatched.* | 4 |
| Primary care appointments | *I was given a telephone diagnosis by a Nurse, and not offered the opportunity to talk to the GP.  The diagnosis was wrong, so I asked for a second opinion.* | 3 |
| Healthcare - associated infection | *My husband had a cystoscopy at our general hospital. He picked up an infection that he still has five months later.  It has not been a good time, but he has been supported by his GP.* | 3 |
| Delayed referral – lack of timely referral of patients | *It took a longer time than expected to be referred to a specialist.* | 3 |
| Wrong diagnosis | *I was misdiagnosed medical issues over the telephone by the GP.* | 3 |
| Vaccine | *My son has a traumatic brain issue as well as other underlying health issues and has still not received his covid vaccine.* | 3 |
| Wrong advice given to patient / caregiver / family members / next of kin | *My wife was not given any specific warning about side effects but was given a leaflet after her vaccine.  She was unwell for four days.* | 3 |
| Transport logistics including transport of patients, documentation, and specimens | *Serious injury and no ambulances due to delays offloading covid patients into hospital.* | 2 |
| Emergency transport – associated with the attendance of an emergency vehicle in an emergency situation | *My mother exhibited classic signs of a stroke. It took over two hours for an ambulance to arrive.  Thankfully it was a TIA [Transient Ischaemic Attack] and she was fine.  In the future, I would drive the person to A+E rather than call an ambulance as it’s only 20 minutes away.* | 2 |
| Complaint/Coroner investigation. Use of the reporting system to alert of complaint or coronial investigation | *A formal complaint was made to the health board at the time. There is insufficient space here to provide the details.* | 2 |
| Missed diagnosis | *The diagnosis was missed by the GP surgery and they failed to refer to a Consultant which led to my husband getting sepsis and he died.* | 2 |
| Adverse drug event – reaction to vaccine | *My wife had an allergic reaction to her covid jab and was given poor advice and medication in A+E.* | 2 |
| Implementing laboratory investigations –issues with the process of obtaining or processing a laboratory specimen | *Blood test for my daughter, struggled to access veins, unsafe practice of manoeuvring the needle in the arm several times, rather than re-attempt.  Plus moved across the room for supplies whilst the needle was still in the arm. This was painful for my daughter and an insufficient specimen was received, resulting in a repeated test needed.* | 2 |
| Issues in the process of investigating a patient’s condition | *I phoned my GP practice to request a blood test and a medication review, which were both due. I was told that the practice is not doing such reviews or blood tests.* | 2 |
| Delayed Assessment - a delay in assessment for care or care adjunct | *I received burns to my face and chest from boiling water, I was left for three hours in pain waiting for an ambulance, and then in A+E I had to wait five hours with no treatment for my burns and was left with no pain relief for the whole time.* | 2 |
| Pressure ulcer developed | *My mother contracted covid in hospital,,, she recovered but came out of hospital completely immobile, unresponsive and covered in bedsores…* | 1 |
| Ability to access out of hours | *Went around in circles trying to speak to somebody in the out of hours service.  Process took almost two hours.  Dropped off at emergency department Wednesday night, they were still in there on Friday morning.  Later was told, that because they had been previously triaged by the out of hours service, they should have been seen earlier.* | 1 |
| Delay in formulating or operationalising a care plan | *As my mothers dementia has worsened during shielding, along with her sight and hearing, we have been unable to get a care plan set up or any assistance at all, despite her living alone.* | 1 |
| Failure to respect patients / caregiver wishes including advanced decisions | *Attending A+E with my elderly mother, and despite her wishes, not able to visit her or in the care home which greatly impacted on her mental health as well as her own….* | 1 |
| Medical records – incorrect documentation or availability of medical records | *Diagnosis and patient advice was mistakenly given by a locum HCP [Health care professional], due to another patients confidential notes being incorrectly attached to this patients file. Patient’s identity was not enquired about nor confirmed by locum during the appointment. Mistake was uncovered by the patient the day after the appointment.* | 1 |
| Referral not performed when indicated | *I should have been consultant led throughout my pregnancy, however this was not picked up until I was over 30 weeks pregnant, community midwives failed to complete the correct paperwork to refer me and failed to send my notes to the hospital.* | 1 |
| No follow up arranged – did not follow-up patient or were not asked to follow-up | *Follow up appointments still not received and do not have an accurate diagnosis.* | 1 |
| Identifying at risk patients – errors in the process of identifying vulnerable patients or patients at high risk | *Our auntie was elderly and in a care home.  During the pandemic she became withdrawn, refusing to leave her room and became depressed.  GP did not visit and diagnosed and prescribed depression over the telephone. We feel that this fell short of the care she should have had. Her death certificate will say old age, but we believe she has died of neglect.* | 1 |
| Insufficient treatment given | *Catheter was not put in correctly, but staff would not return to rectify the issue.* | 1 |
| Contraindicated vaccine | *My mother in her 90s, had contracted covid and self-isolated. We asked the GP surgery if she could get the vaccine after this and they told us it would have be after 28 days, but three days later the district nurses gave her the vaccine.  Days later she became unwell and was hospitalised.* | 1 |
| Ordering laboratory investigations – wrong test ordered or test not ordered when appropriate | *No blood testing for diabetes. I was previously diagnosed as pre-diabetic and used to have regular blood tests, but these have stopped.* | 1 |
| Delay in undertaking appropriate imaging investigations | *I was concerned I may have had cancer.  I was referred to a hospital in Wales for an USS but no appointment arrived after 11 weeks.  I phoned the department but was told that the appointment will not be given for some time as it was deemed routine. I then contacted my specialist at the English hospital and I was given a scan and MRI the next week.* | 1 |
| Responding to results of other investigations – inappropriate response to a result of other investigations | *Was discharged from hospital with a newborn, prior to the newborn blood results due to two blood samples being lost.  Baby came back as DCT [Direct Coombs Test] +ve and could have been very ill. Daily blood tests had to be started immediately, and an internal investigation raised. Baby was not diagnosed and assessed until 72 hours after being born.* | 1 |
| With patients – miscommunication between physicians or healthcare professionals and patients | *I have a hearing impairment so struggle to properly hear what is being said to me, and had difficulties not being able to lip-read. My husband was also not allowed to stay with me, so could not assist.* | 1 |
| Miscommunication between healthcare professionals | *The pharmacy and GPs have not communicated very well with each other, so we are never sure when my husband’s medication will be available and I have to make extra trips to sort it out.* | 1 |
| Equipment / therapeutic adjunct provision –relating to the provision of therapeutic adjuncts | *I was an inpatient in hospital, and a trained nurse was taking observations using a blood pressure machine – she checked six patients and did not clean the machine once between patients.  I challenged and told her not to take mine until she had cleaned it.* | 1 |

**Appendix 5. Nature of patient-reported safety incidents meeting the NHS definition at 18 months**

| **Incident type** | **Example patient narratives** | **Frequency** |
| --- | --- | --- |
| Ability to access physician/healthcare professional – patient delayed or unable to see/speak to physician or HCP or patient | *My sister came to stay and became unwell. She is asthmatic and her breathing was bad, and her oxygen saturations were below 90%. Our GP practice told her that they would not see her as a temporary patient: so, she had to wait for the evening and out of hours service…* | 93 |
| Medication / Vaccine related incidents | *I was prescribed meds for high blood pressure by the GP without seeing him or any discussion as to why it had gotten high. The main side effect was a condition that I already suffer from, completely inappropriate.* | 12 |
| Primary care appointments | *Access to GP and diagnostic services is dreadful, matter now resolved privately at great cost. GPs need to see patients face to face to prevent privatisation, 999 calls, pressure in A+E and Ambulance services.* | 11 |
| Emergency transport – associated with the attendance of an emergency vehicle in an emergency | *Mum was having a heart attack and the ambulance would have taken hours and hours, so dad took her, thankfully she is ok now, but if she had waited, the consultant said she would have died…* | 10 |
| Diagnosis and assessment of patients | *All of my consultations including to the GP have been by phone. A proper assessment cannot take place i.e., a physical exam. HATE HATE HATE THESE USELESS TELEPHONE CONSULTATIONS, HATE THEM.* | 10 |
| Communication failures or breakdowns of communication | *My daughter was admitted as an inpatient leaving her baby at home. The continuity of care she received was very very poor. No one seemed to know what was happening to her and why. She rang me very upset on more than one occasion because no one seemed to care for her needs and she was left in considerable pain, with little or no communication from the team.* | 10 |
| Missing data: Insufficient detail within the free text to determine what is being described. | *-* | 8 |
| Vaccine | *No priority given for my partner to have the Covid vaccine, despite being on Chemotherapy.* | 7 |
| Process of investigating a patient’s condition | *There was a concern that I may have cancer, and the access to testing was delayed by four months due to lack of availability/long waiting lists. This could have been extremely detrimental if I did have cancer.* | 7 |
| Managing appointments for healthcare | *Patients before me at surgery had booked appointments and then did not turn up, this means that others could not be seen. Time to get the message out, that if you book but can’t come then please cancel and make space for others who do want the appointment – don’t be selfish with such a finite resource.* | 6 |
| Delayed diagnosis | *A relative developed pain and swelling at their inner leg. On attending A+E (which was very busy) he was told it was probably phlebitis and given antibiotics and sent home. No scan. Professionals within family suspected it was a DVT, so he returned two days later to find it was a DVT. This delay could have cost him his life.* | 6 |
| Patient fall | *Elderly relative, fell over beside her bed and the staff left her lying, due to health and safety rules…* | 5 |
| Wrong diagnosis | *Wrong diagnosis – bad eye infection instead of removing the foreign particle, meant three visits to hospital and four car shares – these could have led to Covid as I am shielding and do not usually go anywhere.* | 5 |
| General investigations – routine laboratory investigations | *Unable to access regular blood tests, unable to get advice for helping with problem or regular reviews with specialists.* | 4 |
| Incorrect or inefficient transfer of patient for follow-up e.g., requirements for follow up screening or re-review | *Possible cancer lesion on kidney not checked since pandemic began, but just had phone call. Scan was arranged for August but cancelled. Supposed to be now in November (fingers crossed).* | 3 |
| Discharge planning  Includes premature discharge and poor discharge planning e.g., patient discharged home when not appropriate or safe to do so | *My stepdad was diagnosed with prostate cancer and was in the final stages, he kept getting discharged home to my mum who is [elderly]) unsafely. This happened 5 times in 4 weeks…They also tried to discharge him with covid to my clinically vulnerable mum until I stepped in and got social services involved. He died a few weeks later.* | 3 |
| Wrong medication  Incorrect medication dispensed | *Given wrong medication, signed twice saying that it had been checked but was wrong medication with my medication sticker on. This is a never event.* | 3 |
| Healthcare -associated infection | *Bank nurses on night shift were told to create a sterile field before accessing my central line. Some didn’t and as a result contaminated my central line with staph epidermis causing septicaemia again, which necessitated the central line being replaced and me having to stay an extra 2 weeks for IV antibiotics.* | 2 |
| Transport logistics including transport of patients, documentation, and specimens | *Elderly person with pain in their leg, time was taken to diagnose and then hours for an ambulance to be dispatched. The lady had a broken knee resultant of care when hoisted by care worker.* | 2 |
| Inaccurate or unclear medical records | *Hospital doctor mixed up my records with another, seemed to only have the vaguest idea of my condition and simply waffled when asked direct questions…* | 2 |
| Insufficient assessment | *Extremely severe nosebleed, long wait in both ambulance and A+E ward, large quantity of blood taken from my arms, reasons unexplained. Said no - one available to see me or help in anyway. I was left unattended for three hours, and then told to go home. Arm where blood taken bruised from my wrist up to my shoulder for a week.* | 2 |
| Treatment & Procedure related (excluding medication/vaccines) | *Triage stated I would be x rayed, and my ring cut off, I was neither x - rayed and nor was my ring cut off my finger, but I had to go to a hospital the next day for the ring to be cut off due to severe swelling.* | 2 |
| Prescribing medication – wrong medication or wrong dose of medication prescribed, or medication not prescribed when appropriate | *Wrong medication prescribed for my mother, and no awareness from the staff about what she should/shouldn’t be having.* | 2 |
| Dispensing medication orders – issue within the process of delivering a medication order or inappropriate medication order | *Dispensing issues with my medication -again.* | 2 |
| Adverse drug event – patient suffered a complication as a result of medication | *Suffered a severe adverse reaction to second [name of] vaccine and ambulance had to come out. 111 were useless and GP was rubbish too.* | 2 |
| Equipment / therapeutic adjunct provision –relating to the provision of therapeutic adjuncts | *Difficult to get the specialist equipment I needed whilst in hospital.* | 2 |
| Cancer diagnosis delayed | *Misdiagnosed cancer, it was diagnosed too late.* | 2 |
| Vulnerable adults – issues concerning vulnerable adult with no incident identified | *One dementia patient who had a hip replacement frequently tried to walk unaided. She often sat on the bedhead, and if she had fallen the consequences could have been severe. It wasn’t until the day before she was discharged that someone was able to sit with her 24/7.* | 1 |
| Complaint/Coroner investigation. Use of the reporting system to alert of complaint or coronial investigation | *I needed to raise a complaint about my care.* | 1 |
| Secondary care appointments | *A consultant discharged me from their list without looking into my issue because they decided because I had had to re-arrange my appointment that I didn’t deserve to come back. I had to re-arrange because I was ill, and as per their own guidelines would not have been allowed to attend the hospital. It seemed like they were eager to get as many off the list as possible, rather than look at individuals.* | 1 |
| Transfer of patient Information – incorrect or inefficient transfer of patient information across healthcare systems | *Emergency admission of my elderly father. Great difficulty in contacting the hospital. Nurses were helpful when they were able to answer the phone, but impossible to get to the root of the problem. Discharge notes still not reached the GP 10 days later, so no idea really why my father received the treatment that he did.* | 1 |
| Environmental hazard | *A+E in [name of hospital] was filthy, dirty floor, the cleaner swept the rubbish into a pile and then left it to be kicked about by people walking into the department.* | 1 |
| Lost medical records | *The GP was initially dismissive or my problem and did not want to listen to me. When I explained my symptoms, she arranged for bloods and an ECG. The ECG was lost and there was no attempt made to apologise for this or re-arrange the ECG...* | 1 |
| Delayed referral – untimely referral of patients or referral not done when indicated | *An urgent referral to a neurologist wasn’t done, this may not have been forgotten if I hadn’t been just a voice at the end of the phone. This meant a delay of over a month, concerning given my symptoms.* | 1 |
| Referral refused | *Spoke to GP on phone then visited in person for severe and different headache and visual disturbances. He sent an urgent referral to the local hospital who wrote back to say it was probably a migraine and enclosed an information leaflet. They would not see me despite further GP request. I have had migraines for years and these headaches were NOT migraine and GP agreed…* | 1 |
| Social work referral issues | *My 92-year-old mother, who has dementia, is virtually blind and deaf, has arthritis and a heart condition. She has been denied help from social services who prefer to leave it my sister and myself. Both of us are pensioners and I am a cancer patient.* | 1 |
| Referral along diagnostic pathways | *GP failed to follow pathway and sent referral to the wrong department for the wrong procedure until I chased it up and was then referred by the Consultant to the correct department as per the proper pathway.* | 1 |
| Diagnosis | *Respiratory and neurological teams making little effort to look into and diagnose my ongoing problems…. Chest CT cancelled then reinstated by consultant, but then cancelled by radiologist. Pain in chest undiagnosed but ongoing…* | 1 |
| Delayed Assessment: A delay in assessment for care or care adjunct | *Chest CT cancelled and then re-instated by consultant and then cancelled by radiologist, pain in chest undiagnosed and ongoing.* | 1 |
| Clinical treatment: decision errors in the treatment decision-making process | *I came off my bicycle, broke my humerus at shoulder and damaged elbow. Dressing put on without cleaning caused cellulitis which caused awful swelling. Difficult to see the GP Surgery nurses, who finally saw me after I cried on the phone. Needed large doses of antibiotics for 10 days. This experience was very frightening.* | 1 |
| Insufficient treatment / care / monitoring given | *Relative unwashed for many days as an inpatient, never had toothpaste opened, never shaved although electric razor provided, no help to feed, walked into hospital but became malnourished, immobile with multiple pressure sores within a short while. We weren’t allowed to visit more than once a week. He was neglected in [Name of hospital] which is sad because I’m a nurse and wouldn’t have thought I would speak badly about the NHS. Went to wash his laundry that we had been given and mounds of faecal matter had been wrapped up in his clothes and given to us, it fell on to our kitchen floor.* | 1 |
| Timeliness of treatment other than medication – not administered in a timely fashion | *No operations being undertaken; therefore, condition was getting worse – negatively impacting mobility, mental health and increased pain and impacting negatively on other pre-existing medical conditions.* | 1 |
| Adverse drug event – reaction to vaccine | *I developed five days of continuous ocular migraines four days after having my second [name of] vaccine. Having waited 5 hours in A+E, I walked out as I was unable to access a drink and felt worse for the experience.* | 1 |
| Contraindicated medication prescribed for patient – prescribed a drug contraindicated by patients medical or drug history | *I was prescribed a medication over the phone which caused a stomach bleed. I had advised the doctor of issues with my stomach but was prescribed Celoxib. When I had blood in my stool, I tried to get an appointment with a real doctor but instead had to full in an online form, because the surgery was not answering their phone. When surgery heard about blood in my stools, they saw me straight away and sent me to the acute medical unit. I have been prescribed omeprazole for four months and need a magic eye next week.* | 1 |
| Wrong advice – wrong advice given, or no advice / counselling given when appropriate | *When asked, the nurses in the ward were not sure what the safety procedures were for visitors to the ward.* | 1 |
| Implementing laboratory investigations | *The nurse put the needle into my tendon and not my vein when taking a blood test.* | 1 |
| Responding to diagnostic imaging results – inappropriate response to a laboratory result | *Translated from Welsh: The department within the hospital had not informed my sister of what had been seen in the scan, no appointment had been made to investigate it further. It’s as if it is missing in the system.* | 1 |
| Other investigations | *My smear test was delayed by 2 years. This could have been fatal because early detection of cancer is essential. Ironically my annual mammograms took place as scheduled at the local breast hospital.* | 1 |
| Miscommunication between physicians or healthcare professionals and patients | *“My mother is somewhat deaf and finds it impossible to explain her problem or communicate to a GP over the phone.”* | 1 |
| Wrong advice given to patient / caregiver / family members / next of kin | *Partner had DVT after vaccine…Poor advice about second COVID vaccine (told to have [name of vaccine] again). GP at vaccination centre corrected this and advised [other vaccine] due to concern about having a stroke if had first DVT. No communication from hospital team as to what to do next after 3 months of anti-coagulation treatment…* | 1 |
| Equipment related incident | *Placed in a Covid positive waiting area despite not having Covid due to a faulty thermometer being used on arrival.* | 1 |
| Insufficient supply within healthcare | *Blood test was cancelled due to a shortage; results of tests were not combined to achieve a clearer picture of the problem and so tablets were given to mask the problem rather than investigate it further.* | 1 |

**Appendix 6: Factors contributing to patient safety incidents reported at 18 months**

| **Theme** | **Example patient narratives** | **Frequency** |
| --- | --- | --- |
| Covid related (including safety concerns about social distancing/infection control) | *Rather lax attitude to proper use of PPE [personal protection equipment] by health professionals when dealing with patients. Lack of ventilation in crowded waiting rooms.* | 60 |
| Access to GP | *My dad is in remission from non-Hodgkin’s lymphoma. Lives alone and has other health issues. Is supposed to be on GP “hot list” i.e., if he calls the GP, he should be seen in person…. he phoned and was unable to see GP. Had a phone call from GP after several attempts, but this has not re-assured him.* | 36 |
| Access to Healthcare Professional | *Unable to see the doctor when I needed them…* | 34 |
| Accident and emergency department (wait and infection control) | *I nearly choked on a duck bone. Whilst ambulances were great and triage, I was in hospital for nearly four hours+ waiting to be seen and when I was coughing and vomiting, I was studiously ignored by nearby staff.* | 32 |
| Ambulance waiting times | *I was unwell at home, seven weeks after a heart attack. There were no ambulances available despite having symptoms of another heart attack. When I got to A+E there were ambulance queues at the hospital, could not transfer into A+E care because there was no bed available…* | 15 |
| Medication concerns (including repeat prescribing) | *GP failed to see my mother in person, sent her to A+E and urgent care, and then prescribed the incorrect antibiotics and painkillers twice…* | 13 |
| Blood tests not carried out and other investigations | *My father was due to have bloods taken, but when he went to the GP, no one was able to take them…he was told to travel to hospital 10 miles away to have them – if he had been unable to travel this would have caused a delay and anxiety.* | 9 |
| Negative experience of using NHS | *I cannot put into words the catastrophic effect on myself and my family about how my husband was rejected and abandoned by primary care in Wales.* | 8 |
| Expectations of patient with NHS care | *The department was overwhelmed. There was no quiet place, and we were pestered by drunks. Even a letter saying we should be prioritised was completely ignored by triage. Eventually we gave up and went private.* | 7 |
| Covid vaccine | *I struggled to access covid vaccination for my housebound elderly neighbour [elderly], I had to argue their case hard…* | 7 |
| Communication regarding results | *Tested for gestational diabetes, which I had, but was not informed of this for 11 weeks until I had the second test. They had “forgotten” to ring me…* | 6 |
| Telephone- triage related concern | *My daughter was in severe abdominal pain. No face-to-face consultation offered, doctor spoke to her and prescribed over the phone.* | 6 |
| Communication difficulties during appointment | *My mother is deaf and cant explain her problem or communicate with the GP over the phone.* | 5 |
| Delay in management | *Not contacted for surveillance scans or cancer marker blood tests, when contacted consultant was unaware that tests had not been requested …this has delayed my next appointment.* | 5 |
| Fall-related | *My mother fell and was advised by 111 to go for an X-Ray. She waited hours in A+E on a hard metal chair with no communication from staff before deciding to leave due to the pain she was in. She was advised to contact her GP but couldn’t get through, only to be told several days later that she needed to go back to A+E for an X-Ray.* | 5 |
| Access to 111 | *Crisis with my mother at a weekend and NHS 111 was unable to respond for 14 hours due to pressure of work.* | 4 |
|  |  |  |
| Access to Dentist | *I had an infected tooth, there were no dentists available, tried buying a home filling kit to fill the tooth myself…* | 4 |
|  |  |  |
| Family mitigated harm | *My husband had a stroke, I called 999 as we thought it was a heart attack due to the pain in his chest, they couldn’t send an ambulance, so I had to drive him to A+E. He had a massive stroke and collapsed as soon as we walked into the department.* | 4 |
|  |  |  |
| Medication dispensing concern | *Medication that is taken daily was not available from pharmacy. When it was available, I was given two months of medications at once, and because we foster children, having large volumes of medication in the house is a risk.* | 4 |
| Policies/staff not following policy | *Concern about my own safety as an inpatient due to covid, due to staff not following their own infection control policies.* | 4 |
| Staffing levels insufficient | *In an acute orthopaedic ward, elderly patients with dementia were left unattended due to staff shortages and a failure to follow safeguarding procedures.* | 4 |
| Access secondary care appointment | *My son was diagnosed with [condition] in his eye during the pandemic. This diagnosis was preceded by an 18 month wait from the original referral, which was marked as routine (12 month wait pre-covid, plus 6 months further due to covid). Since then, there has been a further delay and lack of communication between eye specialists. I suspect this reflects NHS understaffing issues that pre-existed the pandemic.* | 3 |
| Child - related issues | *My child was not triaged for a head injury for over four hours.* | 3 |
| Collecting medication difficulty | *Turned up to practice to collect medication, despite waiting for ages without social distancing, they told me that medication was not there, and I had to go to pharmacy.* | 3 |
| Social distancing concerns | *My GP waiting room has poor ventilation and is too small for social distancing. I am not comfortable being there or touching the touch screens to book into my appointment.* | 3 |
| Access to Physiotherapist | *Phoned to explain that I had trouble brewing and needed physio, was told that they would get back to me…but they didn’t.* | 3 |
|  |  |  |
|  |  |  |
|  |  |  |
| Access to SALT and OT/Community Paediatrics | *Needed appointment with OT [Occupational Therapist], nothing available despite leaving multiple messages.* | 2 |
| Access to Nurse | *Unable to see the nurse due to concerns over a spot on my chest.* | 2 |
|  |  |  |
| Equipment error within hospital | *The wrong piece of equipment was used during the procedure.* | 2 |
|  |  |  |
| Allergic reaction reported | *Suffered an adverse reaction to second [name of] vaccine, and ambulance had to come out. 111 were useless and GP was rubbish too…* | 2 |
|  |  |  |
| Referrals | *An urgent referral to a neurologist wasn’t done, this may have not been forgotten if I had not just been a voice at the end of the phone. This has meant a delay of over a month, concerning given my symptoms.* | 2 |
|  |  |  |
|  |  |  |
|  |  |  |
| Discharge from hospital concern | *We were not able to visit and were not pleased with the standard of care. My mother was discharged with Clostridium. Difficile, in flimsy clothes and no underwear (she was incontinent) in March. She was also not given any instructions for taking her medication and had a morphine patch even though the hospital said she as not prescribed morphine. This made her sleepy and we could not work out why, she could have had a fall.* | 1 |
|  |  |  |
| Record error | *It took me weeks to get my past records corrected and I cannot trust the inpatient care when I am next admitted to hospital, it’s like Russian Roulette.* | 1 |
|  |  |  |
|  |  |  |
| Wrong treatment | *Chaos in A+E, no identity band until discharge, confused instructions RE nil by mouth which led to dehydration and confusion – lots of contradictory information given.* | 1 |

**Appendix 7. Nature of patient-reported safety incidents meeting the NHS definition at 24 months**

| **Incident category** | **Incident type** | **Example patient narratives** | **Frequency** |
| --- | --- | --- | --- |
| Managing appointments  (63) | Healthcare setting not named | *My husband had a memory test. Usually, it is recommended that someone they know attends with them, but because of NHS ultra-caution, it was not possible. Therefore, I believe that his problem is not being taken seriously enough and am very worried.* | 35 |
|  | Primary care | *My GP has refused to see me face to face despite continued fainting episodes and a 5-month long period.* | 21 |
|  | Secondary care | *I’ve had 4 cancelled appointments in the last 2 months for a gynaecology appointment. I have severe endometriosis and recurrent miscarriage and been offered no support. My GP was very dismissive when contacted it’s extremely traumatic and has affected my mental health.* | 7 |
| Ability to access healthcare professionals  (39) | Ability to access healthcare professionals | *If I could have accessed a GP appointment I would not have had to wait in A&E for 20 hours. Treatment could perhaps have been started earlier.* | 34 |
|  | Out of hours | *There was confusion around 111, CAV24/7, A&E, GP and other units- in who should treat me, whether I needed treatment, and the processes to follow. I was sent in circles for a couple of days before finally being seen at a minor injuries’ unit.* | 5 |
| Emergency transport  (25) | Emergency transport – errors associated with the attendance of an emergency vehicle in an emergency | *My husband collapsed with chest pain on 3 separate occasions. Each time he lost consciousness. I was put on hold when calling 999. The first time the ambulance arrived 6 hours later, the second and third time I was told there was nobody available and had to take him to A&E myself. This was difficult physically and very scary.* | 25 |
| Covid related  (24) | COVID-related | *Of the six or seven people I dealt with at close quarters on the day of the operation, three had their masks below their noses - including the guy delivering food. this may seem minor, but infection levels were high and my catching it mid breast cancer surgery with more treatment to come would not have been good.* | 24 |
| Medication and Vaccines  (28) | Prescribing | *The deal was having an emergency appointment and treatment required for my husband with health issues. Wrong prescription prescribed for pre-treatment antibiotics and medication being already taken.* | 15 |
|  | Vaccine-related | *Severe delays attempting to access my husband’s first booster. Centre was difficult to reach, involved parking on a narrow country road and despite having an appointment, we waited for over two hours and we’re still nowhere near the front of the queue. We then had to leave, as my husband has incontinence issues and there was no disabled toilet. We returned on Sunday and there were very few patients, but the nurse could not log into her computer and then had great difficulty drawing up the injection, spilling a quantity.* | 8 |
|  | Dispensing | *Pharmacy saying, they had ordered repeat prescriptions, missed half of items causing multiple visits to pharmacy, stress, missing treatments, and therefore multiply exposure to Covid.* | 5 |
| Treatment and Procedure  (7) | Insufficient treatment | *Lack of care on hospital ward. Admitted with Urinary tract sepsis. Was placed on respiratory ward with staff who had no knowledge of urology. Was left 10 days with sheath catheter that should be changed daily as no one knew how to change it.* | 7 |
